# Impact of gut permeability on the breast microbiome using a non-human primate model

**DOI:** 10.1017/gmb.2022.9

**Published:** 2022-11-09

**Authors:** Alaa Bawaneh, Carol A. Shively, Janet Austin Tooze, Katherine Loree Cook

**Affiliations:** 1 Department of Surgery, Wake Forest University School of Medicine, Winston-Salem, NC, USA; 2 Integrative Physiology and Pharmacology, Wake Forest University School of Medicine, Winston-Salem, NC, USA; 3 Department of Pathology, Section of Comparative Medicine, Wake Forest University School of Medicine, Winston-Salem, NC, USA; 4 Department of Biostatistics and Data Science, Wake Forest University School of Medicine, Winston-Salem, NC, USA; 5 Department of Cancer Biology, Wake Forest School of Medicine, Winston-Salem, NC, USA

**Keywords:** Western and Mediterranean diet, entero-mammary axis, *Streptococcus lutetiensis*, *Lactobacillus*, 16S sequencing, metagenomics sequencing

## Abstract

We previously demonstrated in non-human primates (NHP) that Mediterranean diet consumption shifted the proportional abundance of *Lactobacillus* in the breast and gut. This data highlights a potential link about gut-breast microbiome interconnectivity. To address this question, we compared bacterial populations identified in matched breast and faecal samples from our NHP study. Dietary pattern concurrently shifted two species in both regions; *Streptococcus lutetiensis and Ruminococcus torques.* While we observe similar trends in *Lactobacillus* abundances in the breast and gut, the species identified in each region vary; Mediterranean diet increased *Lactobacillus_ unspecified species* in breast but regulated *L. animalis* and *L. reuteri* in the gut. We also investigated the impact of gut permeability on the breast microbiome. Regardless of dietary pattern, subjects that displayed increased physiological measures of gut permeability (elevated plasma lipopolysaccharide, decreased villi length, and decreased goblet cells) displayed a significantly different breast microbiome. Gut barrier dysfunction was associated with increased α-diversity and significant different β-diversity in the breast tissue. Taken together our data supports the presence of a breast microbiome influenced by diet that largely varies from the gut microbiome population but is, however, sensitive to gut permeability.

## Introduction

It is estimated that the human body contains more bacterial cells than human cells (Sender et al., 2016). While the majority of the bacteria biomass is contained to the intestinal tract, microbes in lower abundance have been identified in other organ types located distal to the gut, including the breast tissue. The presence of a mammary gland (MG; adipose/stroma/epithelial cells) bacterial population was identified in human breast tissue samples taken from non-lactating, non-pregnant women undergoing reduction mammoplasty, lumpectomy, or mastectomy surgeries (Urbaniak et al., 2014). There were large differences in the type and proportional abundance of bacterial taxa detected between the two geographical populations. Since then, others have also shown normal breast tissue-specific and breast tumour-specific microbiomes to exist (Banerjee et al., 2018; Chiba et al., 2020; Hieken et al., 2016; Nejman et al., 2020; Parida and Sharma, 2020; Plaza-Diaz et al., 2019; Shively et al., 2018; Soto-Pantoja et al., 2021; Thompson et al., 2017; Tzeng et al., 2021; Urbaniak et al., 2014). Another study investigating the microbiome of breast tissue obtained from patients with benign or malignant breast cancers showed that those with malignant tumours displayed a distinct microbiota population (Hieken et al., 2016), suggesting breast tissue dysbiosis as a possible driver of breast cancer. Our group demonstrated breast cancer patients with obesity displayed different proportional abundances of several family-level bacterial taxa in breast tumour tissue, suggesting that obesity may influence the breast microbiome (Chiba et al., 2020).

The concept of a gut-mammary gland signalling axis initially proposed and investigated in the lactation setting, suggests that the gut microbiome may influence the breast microbiome (Fernandez et al., 2013, 2020; Rodriguez, 2014). We recently demonstrated the impact of dietary-induced microbiota changes on breast cancer risk conferred by obesity (Soto-Pantoja et al., 2021). Microbiome transplantation from mice on a high-fat diet to mice on a low-fat diet increased mammary tumour incidence to that of the high-fat diet group in a carcinogen tumorigenesis model. Oral faecal microbiome transplants shifted both the gut and mammary tumour microbiomes. Consumption of a high-fat diet and faecal transplant of lard-derived faecal microbiota increased systemic and MG levels of lipopolysaccharide (LPS), suggesting a potential gut-breast signalling axis. Using breast tumour and normal tumour-adjacent breast samples from a window-of-opportunity clinical trial, we found that dietary interventions, such as omega-3 polyunsaturated fatty acids supplementation, was associated with changes to the tumour and breast microbiome populations (Soto-Pantoja et al., 2021).

Our previous non-human primate (NHP) model showed that dietary pattern (Western vs. Mediterranean diet) can shift the breast microbiome (Shively et al., 2018). Long-term consumption of a Mediterranean diet resulted in a 10-fold increase in breast *Lactobacillus* abundance, with no apparent change in total bacteria biomass. This study was paired with untargeted metabolomics in subject-matched plasma and breast samples to indicate specific breast-localised regulation of bile acid metabolites and bacteria-modified bioactive compounds, suggesting the presence of a modifiable breast-specific microbiome. We have also recently reported that dietary pattern and adiposity shifts the gut microbiome in NHP, in which lean Mediterranean diet-fed NHP display sixfold increase in gut *Lactobacillus animalis* (Newman et al., 2021), suggesting potential similar regulation of certain breast and gut microbiota populations by diet. To determine the dietary interactions regulating the gut and breast microbiomes, we compared the gut and breast microbiome populations in matched samples from NHP dietary cohort. We further explored the influence of a “leaky gut” on the breast microbiome. We now demonstrate that the breast has its own bacterial niche sensitive to diet that is largely different from the gut microbiome population but is influenced by gut permeability.

## Material and methods

### Non-human primate subjects

Adult female *Macaca fascicularis* were obtained (SNBL USA, Ltd. Alice, TX) and housed in groups with daylight exposure on a 12/12 light/dark cycle. Animals were randomised to a dietary pattern [Western or Mediterranean; See reference (Newman et al., 2021; Shively et al., 2018, 2019) for further detail on the model and experimental diets]. Faecal samples were collected from subjects at 26 months. Breast tissue samples were collected at the end of the study at 31 months (*n* = 11–12 subjects per diet). All animal manipulations were performed according to the guidelines of state and federal laws, and the Animal Care and Use Committee of Wake Forest University School of Medicine.

### Metagenomic and 16S sequencing

DNA was isolated from 100 mg of frozen faeces or MG tissue using the Qiagen DNeasy PowerSoil Pro kit protocol. Metagenomic sequencing and 16S sequencing were performed by CosmosID Inc. (Rockville, MD). For further details on metagenomics sequencing please see references (Newman et al., 2021). For 16S sequencing, DNA libraries were prepared using Illumina 16S Metagenomic Sequencing kit (Illumina, Inc., San Diego, CA) according to the manufacturer’s protocol. The V3–V4 region of the bacterial 16S rRNA gene sequences was amplified using the primer pair containing the gene-specific sequences and Illumina adapter overhang nucleotide sequences. The full-length primer sequences are: 16S Amplicon PCR Forward Primer (5′-TCGTCGGCAGCGTCAGATGTGTATAAGAGACAGCCTACGGGNGGCWGCAG) and 16S Amplicon PCR Reverse Primer (5′ GTCTCGTGGGCTCGGAGATGTGTATAAGAGACAGGACTACHVGGGTATCTAATCC).

Amplicon PCR was performed to amplify template out of input DNA samples. PCR product was cleaned up from the reaction mix with Mag-Bind RxnPure Plus magnetic beads (Omega Bio-Tek, Norcross, GA). The library (~600 bases in size) was checked using an Agilent 2200 TapeStation and quantified using QuantiFluor dsDNA System (Promega). Libraries were normalised, pooled and sequenced (2 × 300 bp paired-end read setting) on the MiSeq (Illumina, San Diego, CA). Zymo community standard (D6305) was used as a positive control and lab-grade DEPC (diethylpyrocarbonate)-treated water was used as a negative control. 16S read depth per sample was >30,000 (range: 33,665–106,582 reads).

### Intestinal permeability measurements

Formalin-fixed paraffin-embedded intestinal tissue (colon and ileum) were cut into 5 μm sections and stained using a haematoxylin and eosin (H&E), Alcian blue (Abcam Cat#, ab150662), or mucicarmine (Abcam Cat# ab150677) staining protocol. Staining was visualised by Mantra Quantitative Pathology Image System, 20× objective was used in H&E staining for muscularis thickness measurements and 10× objective for villi length, then images were quantified using ImageJ program (2 pixels/μm, and 1 pixel/μm, respectively). Goblet cells were manually counted per villus using 20× objective. Four representative images from each tissue were quantified and averaged per subject. Snap-frozen plasma samples collected at necropsy were used to measure circulating LPS concentrations by ELISA (LSBio, Cat# LS-F17912) following the manufacturer protocol. NHP subjects regardless of dietary pattern were sub-grouped into LPS high NHP subjects (*n* = 10) that a mean plasma LPS of 125 ± 73 pg/mL and LPS low subjects (*n* = 13) that displayed a mean plasma LPS of 21 ± 9 pg/mL, based upon LPS concentrations of 50 pg/mL (approximately circulating serum levels in healthy human subjects). Two subjects with intermediate LPS plasma levels and were excluded from analysis.

### Statistical analysis

**Figure 1:**
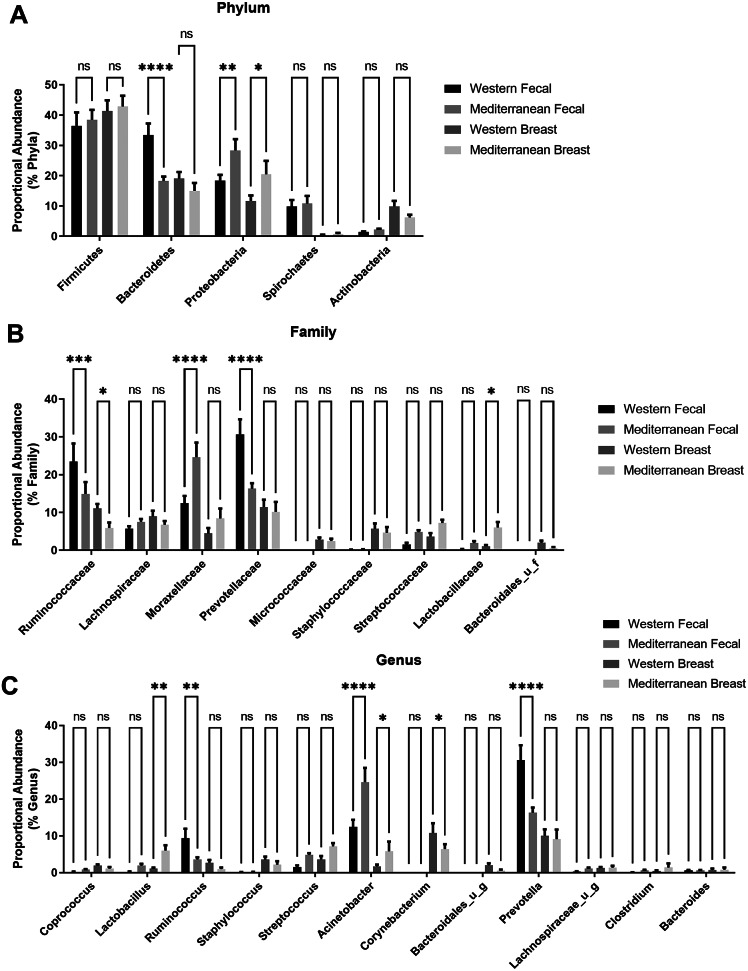
Comparing the proportional abundance of the most abundant microbes between the gut and breast compartments. (A) Phylum classification of faecal and breast bacterial populations shows populations differ by tissue type and diet administration. (B) Family level classification of microbes in faeces and breast samples. (C) Genus level of classification of microbes regulated by diet in faecal and breast tissue. Two-way ANOVA followed by Holm–Šídák’s multiple comparisons test. *n* = 11–12. *p-value < 0.05.

**Figure 2:**
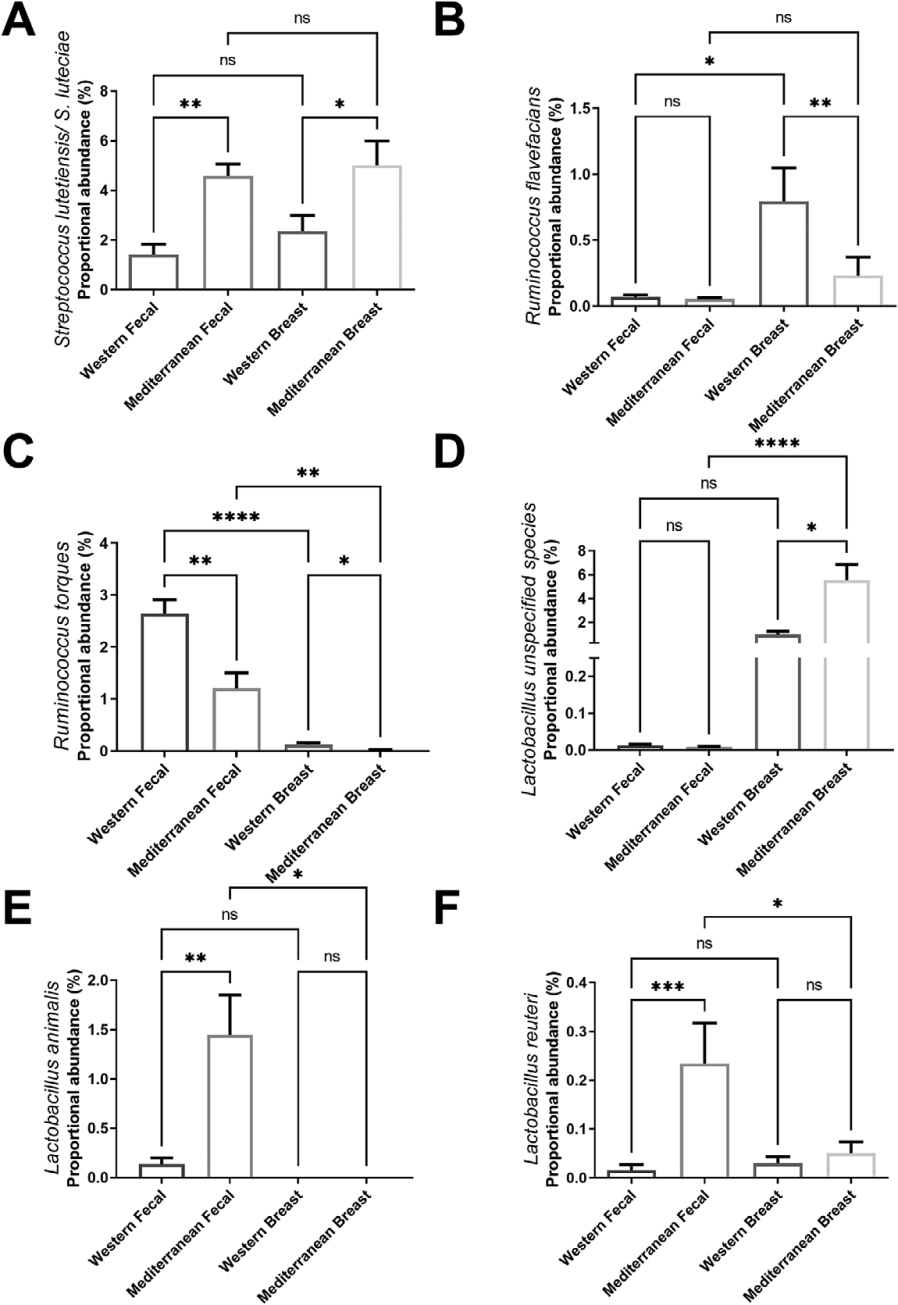
Specific bacterial taxa identified in faecal and breast tissue samples regulated by dietary pattern. (A) Mediterranean diet consumption increased proportional abundance of *Streptococcus lutetiensis* in both the breast and faeces. (B) Western diet-fed subjects displayed elevated *Ruminococcus flavefacians* abundance in the breast tissue, which was unchanged in the faeces. (C) Western diet consumption displayed elevated proportional abundance of *Ruminococcus torques* in both the breast and faeces. (D) Mediterranean diet-fed subjects displayed elevated *Lactobacillus-unspecified species* in their breast tissue, but not their faecal samples. Mediterranean diet consuming NHP displayed elevated proportional abundance of *Lactobacillus animalis* (E) and *Lactobacillus reuteri* (F) in the gut but not in their breast tissue. *n* = 11–12. *p-value < 0.05. Kruskal Wallis test with Dunn’s post hoc analysis.

**Figure 3:**
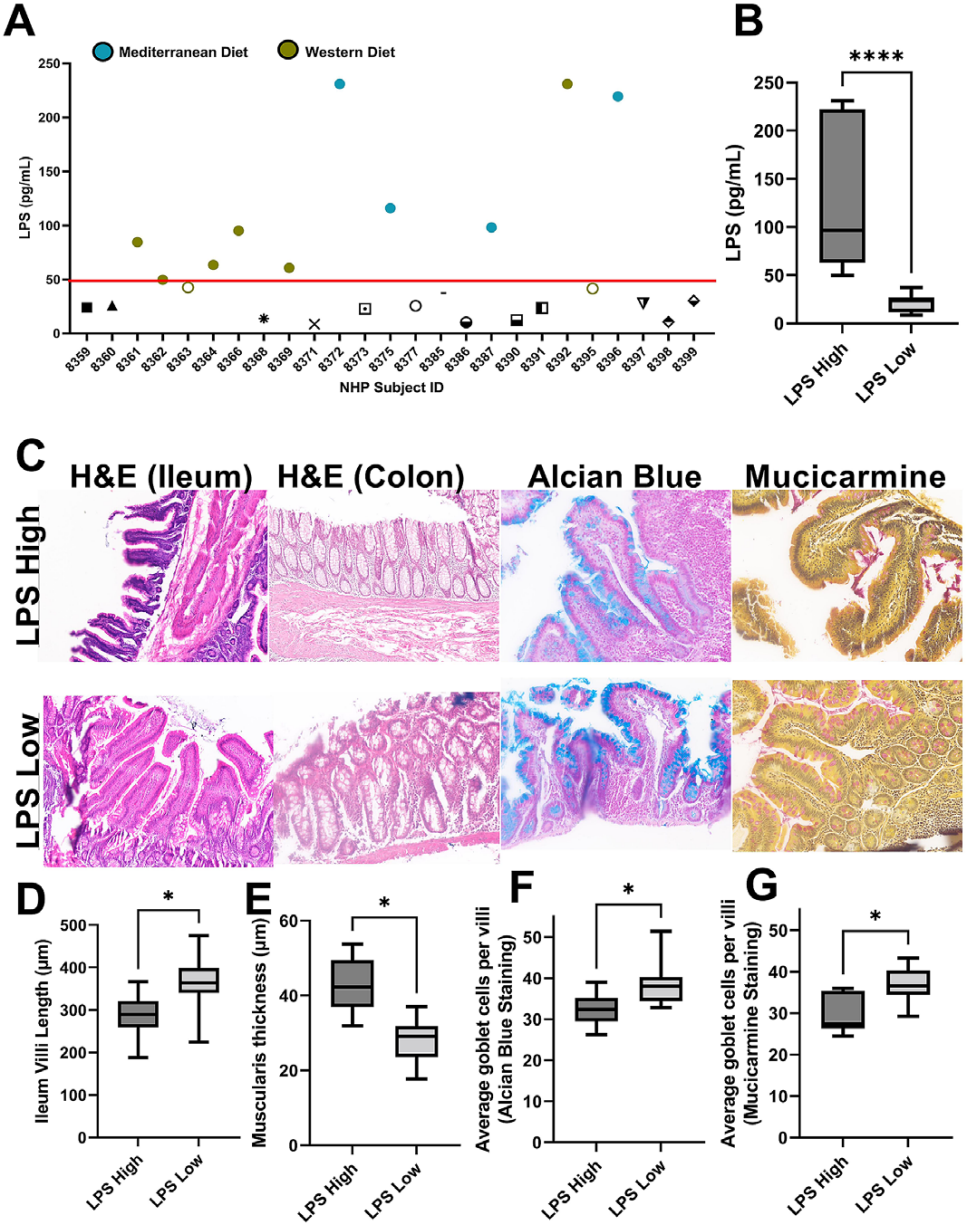
NHP subjects can be sub-grouped by intestinal permeability markers. (A) NHP subjects in which matched faecal and breast microbiome sequencing was performed were analysed for circulating plasma lipopolysaccharide (LPS) by ELISA. Red line demarks LPS concentration of 50 pg/mL (approximately circulating serum levels in healthy human subjects). Teal-filled circles are Mediterranean diet-fed subjects with high LPS (*n* = 4) and chartreuse-filled circles are Western diet-fed subjects with high LPS (*n* = 6). The two chartreuse unfilled circles are subjects with intermediate LPS plasma levels and were excluded from analysis. (B) Regardless of dietary pattern, LPS high NHP subjects (*n* = 10) displayed a mean plasma LPS of 125 ± 73 pg/mL which was significantly higher than the mean LPS (21 ± 9 pg/mL) observed in the LPS low subjects (*n* = 13). ****p < 0.0001. Intestinal health measurement including villi length, muscularis thickness, and goblet cell counts were performed on paraffin-embedded ileum and colon tissue from NHP subjects. Representative images H&E, Alcian blue, and mucicarmine stained tissue is shown in (C). LPS high subjects displayed reduced villi length (D), increased muscularis thickness (E), and decreased goblet cell counts (F,G) when compared to LPS low subjects suggesting decreased barrier function and elevated gut permeability in LPS high subjects. *n* = 10–13; *p-value < 0.05, unpaired *t*-test with Welch’s correction.

**Figure 4:**
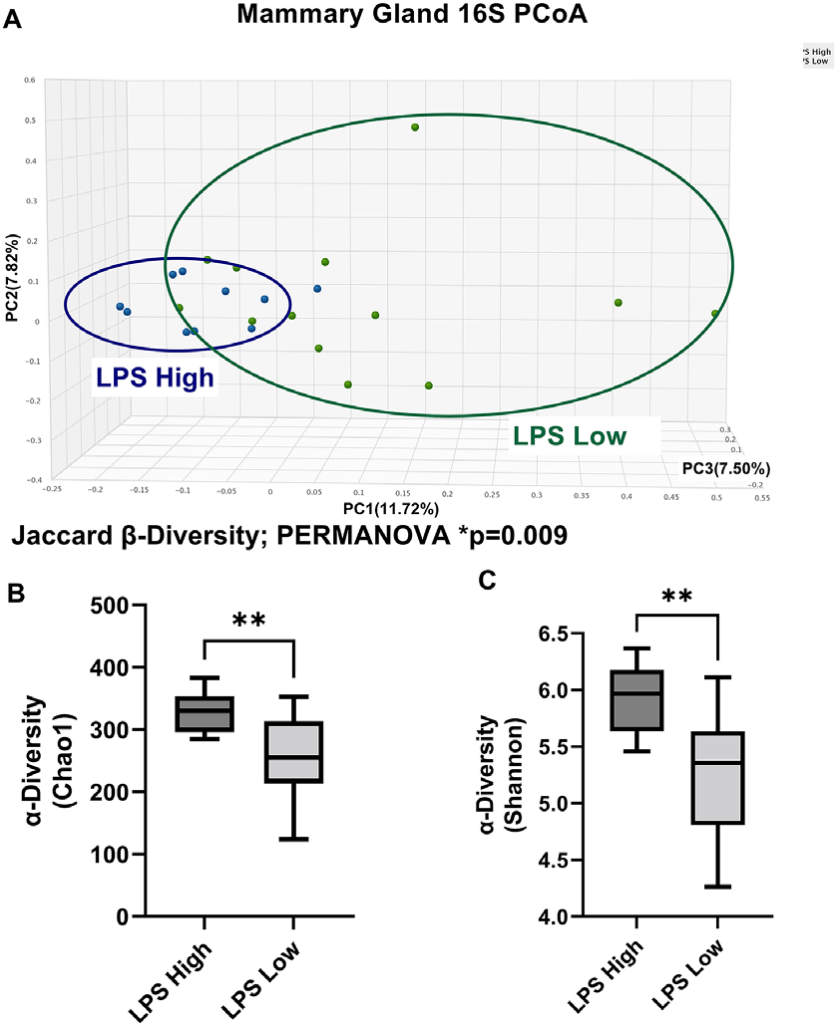
Breast 16S sequencing by plasma LPS levels indicates gut permeability significantly modulates the NHP breast tissue microbiome. (A) β-diversity principal coordinate analysis (PCoA) Jaccard distance demonstrates LPS high versus LPS low NHP subjects display different breast microbiota populations. *n* = 10–13, Permutational multivariate analysis of variance (PERMANOVA) *p*-value = 0.009. Chao1 (B) and Shannon (C) α-diversity is significantly higher in breast samples from LPS high NHP versus LPS low NHP subjects. *n* = 10–13; **p-value < 0.01; unpaired two-tailed *t*-test.

**Figure 5:**
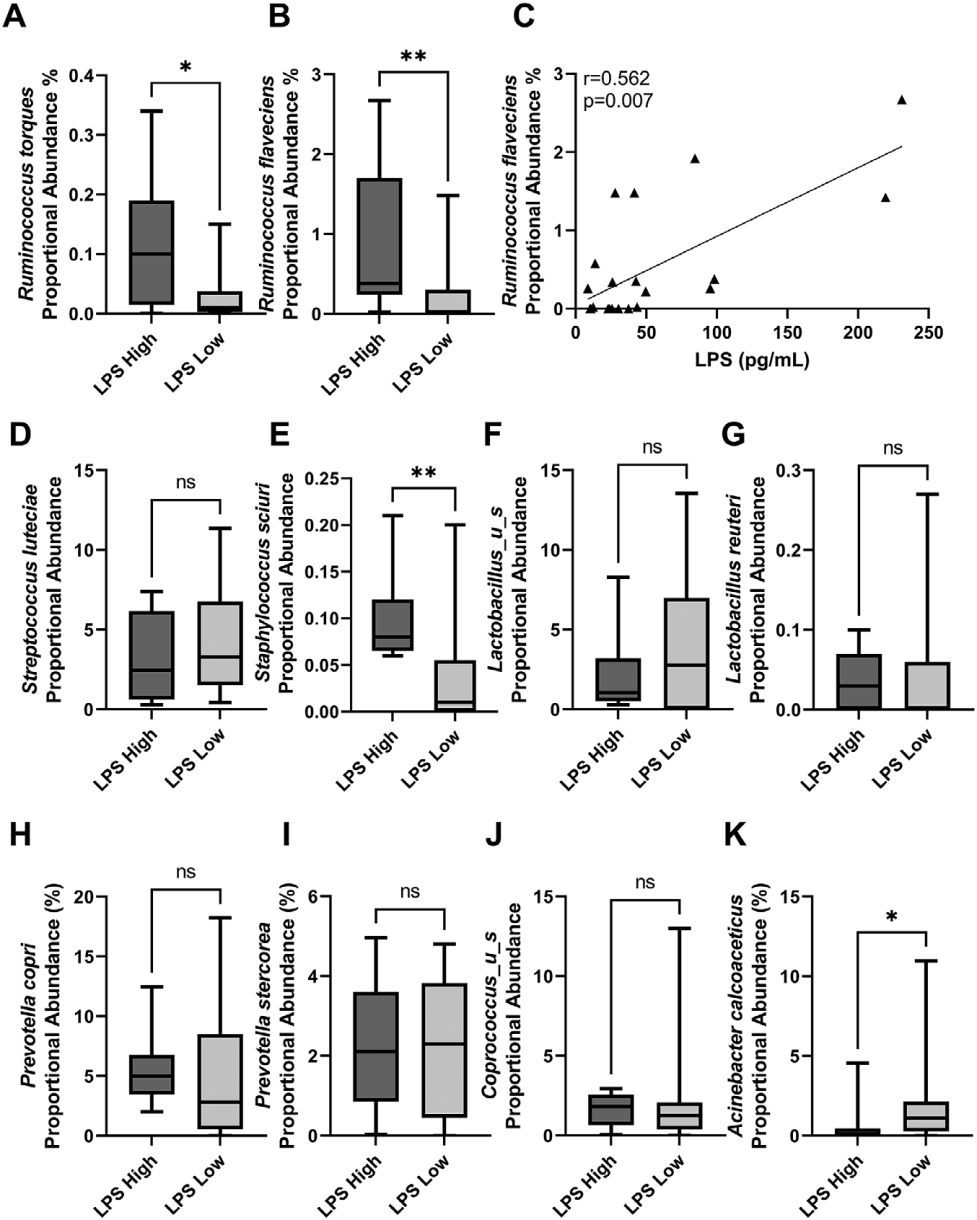
*Ruminococcus* species regulated by diet in breast tissue are modulated by a leaky gut. NHP subjects with high plasma LPS display significantly elevated *Ruminococcus torques* (A) and *Ruminococcus flaveciens* (B) proportional abundance within their breast tissue when compared with NHP subjects with low plasma LPS levels. *n* = 10–13; *p < 0.05, **p < 0.01; non-parametric Mann–Whitney *t*-test. (C) Breast *Ruminococcus flaveciens* abundance positively correlates with plasma LPS concentration. *n* = 23; Spearman’s correlation, *r* = 0.562, *p* = 0.007. Plasma LPS concentration had no significant effect on the proportional abundance of *Streptococcus luteciae* (D), *Lactobacillus_u_s* (F), *Lactobacillus reuteri* (G), *Prevotella copri* (H), *Prevotella stercorea* (I), or *Coprococcus_u_s* (J). Breast samples from LPS high NHP displayed higher *Staphylococcus sciuri* (E). LPS low subjects displayed significantly elevated breast *Acinetobacter calcoaceticus* (K) than LPS high subject breast tissue *n* = 10–13; *p-value < 0.05; non-parametric Mann–Whitney *t*-test.

16S sequencing data were analysed by the CosmosID 16S pipeline and database. Results were presented as an operational taxonomic unit (OTU) table, visualised as heatmaps, stacked bar charts, alpha diversity plots, and beta diversity network graphs. Data are presented as bar plots (Figures 1 and 2) and box plots (Figures 3–5). Permutational multivariate analysis of variance (PERMANOVA) was used for β-diversity PCoA comparison. Wilcoxon rank sum test was performed for α-diversity comparisons. For data in Figure 1, Two-way ANOVA followed by Holm–Šídák’s multiple comparisons test. Non-parametric Kruskal Wallis test followed by a Dunn’s post hoc analysis was used to compare specific bacterial species abundances in faecal and breast samples (Figure 2). Non-parametric Spearman’s correlation was used for LPS and *Ruminococcus flavefacians* associations. Plasma LPS and intestinal pathology comparisons (villi length, goblet cell counts, and muscularis thickness) were assessed using two-tailed unpaired *t*-test with Welch’s correction. A non-parametric Mann–Whitney *t*-test was performed for breast species proportional abundance by LPS sub-groups (Figure 5). *p-value < 0.05 was set for determining statistical significance.

## Results

At the phylum level, gut Bacteroidetes proportional abundance was modulated by diet but not the breast population. Both the breast and faecal samples displayed elevated proportional abundance of Proteobacteria at the phylum level when subjects were consuming a Mediterranean diet (Figure 1A). At the family level, diet only similarly shifted Ruminococcaceae proportional abundance in both the breast and gut (Figure 1B). At the genus level, dietary pattern shifted Acinetobacter (*p*-value < 0.02), Lactobacillus (trend; ≤0.1 faecal, *p*-value < 0.01 breast), and Ruminococcus (trend; ≤0.05 faecal, *p*-value = 0.07 breast) in both the gut and breast tissue microbiome (Figure 1C).

We also identified several taxa specifically regulated by diet in both the breast and gut regions. Mediterranean diet consumption increased proportional abundance of *Streptococcus lutetiensis* in both the breast and faeces (Figure 2A). Western diet-consuming subjects displayed elevated proportional abundance of *Ruminococcus flavefacians* in their breast tissue but not in the faecal samples (Figure 2B). Western diet consumption displayed elevated proportional abundance of *Ruminococcus torques* in both the breast and faeces (Figure 2C). Mediterranean diet-fed subjects displayed elevated *Lactobacillus_unspecified species* (Figure 2D) in their breast tissue, while displaying increased *Lactobacillus animalis* (Figure 2E) and *Lactobacillus reuteri* (Figure 2F) in the gut. Diet did not significantly shift *L. reuteri* in the breast tissue and *L. animalis* was not present in breast tissue. Species-specific localization of *Coprococcus* was observed in the gut and breast regulated by diet (Supplementary Figure S1A). *Coprococcus comes* and *Coprococcus catus* were elevated in the gut of Mediterranean diet-fed NHP but undetectable in breast tissue. Breast tissue of Western diet-fed NHP displayed higher *Coprococcus_unspecified genus*, which was not detected in gut populations. While *Prevotella copri* and *Prevotella stercorea* are present in both tissue compartments, these species only differed by diet in the gut compartment (Supplementary Figure S1B). Species-specific localization of Acinetobacter species in the gut differed by diet (Supplementary Figure S1C), where *A. baumanii/calcoaceticus* is higher in Mediterranean diet-consuming subjects within the gut, but not in the breast.

Microbial dysbiosis often leads to tight junction protein deregulation enabling bacterial translocation and metabolic endotoxemia (Fuke et al., 2019). To determine whether gut barrier dysfunction modulated the breast microbiome, we first measured gut health parameters in our NHP subjects. Plasma LPS concentration was determined in each subject and graphed by individual subject (Figure 3A). Based upon previous human serum LPS measurements associated with metabolic endotoxemia that established an approximate 50 pg/mL LPS as an average control serum concentration (Kallio et al., 2015), we then sub-grouped the NHP subjects regardless of diet into LPS high (*n* = 10) or LPS low (*n* = 13). LPS high NHP subjects’ mean plasma concentration was 125 ± 73 pg/mL compared with LPS low NHP subjects’ mean plasma concentration of 21 ± 9 pg/mL (Figure 3B). We also stained paraffin-embedded intestinal tissue to measure villi length, muscularis thickness, and goblet cells by H&E, Alcian blue, and mucicarmine. Representative images are shown in Figure 3C. NHP subjects within the LPS high designation displayed decreased villi length (Figure 3D), increased muscularis thickness (Figure 3E), and decreased goblet cells (Figure 3F,G). These data indicate that NHP subjects in the LPS high group demonstrate impaired gut barrier function and increased permeability.

We then re-analysed the breast 16S microbiome sequencing results by circulating LPS concentrations to investigate whether impaired gut barrier function may influence the breast tissue microbiome. Breast microbiota in NHP subjects from the LPS high group displayed significantly different β-diversity principal coordinate analysis (PCoA) Jaccard distance when compared with the breast samples from the LPS low group (Figure 4A). The LPS high group also displayed significantly elevated Chao1 α- diversity (Figure 4B) and Shannon α-diversity (Figure 4C) when compared with the LPS low group.

At the species level, breast tissue from NHP subjects in the LPS high group displayed a significantly higher proportional abundance of *Ruminococcus torques* (Figure 5A) and *Ruminococcus flavefaciens* (Figure 5B) than breast tissue from NHP subjects in the LPS low group. While *R. torques* did not significantly correlate with plasma LPS, *R. flavefaciens* abundance positively associates with plasma LPS concentrations (Spearman’s correlation *r* = 0.562, *p*-value = 0.007, *n* = 23; Figure 5C). Other microbe species identified as co-regulated by diet within the gut and breast were not significantly regulated by gut barrier dysfunction groups (*Streptococcus luteciae*, Figure 5D; *Lactobacillus_u_s*, Figure 5F; *Lactobacillus reuteri*, Figure 5G; *Prevotella copri*, Figure 5H; *Prevotella stercorea*, Figure 5I; and *Coprococcus_u_s*, Figure 5J). *Staphylococcus sciuri* was higher in breast tissue from LPS high NHP (Figure 5E). *Acinetobacter calcoaceticus* was significantly higher in the breast tissue from the LPS low NHP subjects (Figure 5K).

## Discussion

The concept of an entero-mammary transmission route as a potential active mechanism to transfer live bacteria from the gastrointestinal tract to the mammary gland through the mesenteric lymph node has been proposed (de Andres et al., 2017; Jimenez et al., 2008; Perez et al., 2007). Pathological conditions that disrupt the gut barrier increase bacterial translocation from the gut to other tissue types, supporting a “leaky gut” model (Cheng et al., 2018; Mokkala et al., 2016; Ortiz et al., 2014). Strictly interrogating our NHP gut microbiome and breast microbiome by dietary pattern consumption suggests that diet independently regulates the breast and gut bacterial populations with few populations expressed in both regions being similarly regulated by diet, diminishing the role of the entero-mammary transmission route for the breast microbiome. However, NHP subjects with increased intestinal permeability did display a significant difference in both alpha and beta diversity, indicating that a “leaky gut” mode of transmission may indeed influence the colonisation or selection of microbes comprising the breast microbiome.

Comparing microbiota populations in NHP subjects randomised to consume a Western or Mediterranean diet, we are able to show that the majority of gut microbiota species are not present in the breast compartment. For the most part, this is unsurprising as the microenvironmental pressures (pH, oxygen content, glucose availability) widely differ between regions. Only two species (*Ruminococcus torques* and *Streptococcus lutetiensis*) are similarly regulated by diet in each compartment, with Western diet consumption correlating with increased *R. torques* and Mediterranean diet consumption associated with increased *S. lutentiensis.* *S. lutentiensis* is a lactic acid-producing, Gram-positive, facultative anaerobe that displays similar proportional abundance and regulation by diet in both the gut and breast regions. *R. torques* is a mucin-degrading, Gram-positive, anaerobe with approximately 20-fold higher proportional abundance in the gut than the breast tissue in Western diet-fed subjects, suggesting dietary patterns influence this microbe similarly in both locations. Since the majority of microbes identified regulated by diet are dependent on body region, this most likely indicates that dietary metabolites in circulation offer selection pressures to modify the bacteria populations already present in the breast tissue. Further research is needed to determine the physiological relevance of the breast microbiome on tissue homeostasis and signalling.

On the other hand, subjects with elevated circulating plasma LPS which is a marker of a gut barrier dysfunction display a different breast microbiome than NHP subjects with low levels of circulating LPS regardless of diet. Metabolic endotoxemia, characterised by elevated circulating level in plasma/serum LPS resulting in chronic low-grade inflammation, is associated with obesity and metabolic syndrome (Boutagy et al., 2016). Studies measuring serum LPS in obese versus non-obese patients report a significant 26% increase in serum LPS in obese patients compared with non-obese patients (Kallio et al., 2015). Increased gut permeability markers in NHP subjects were associated with increased microbial α-diversity and β-diversity PCoA in breast tissue, suggestive of either a gut-breast signalling axis or a potential LPS-mediated selection pressure on present populations. Of the common species present in both the gut and breast compartment only *Ruminococcus torques* were associated with gut barrier dysfunction. *Ruminococcus torques* (a bacterial species categorised within the Firmicute phyla) is anaerobic mucin-degrading bacteria associated with dysbiosis and decreased barrier function in the gut (Cani, 2014; Rajilic-Stojanovic and de Vos, 2014). Elevated *R. torques* is associated with irritable bowel disease, obesity, autism, and circadian rhythm disruption (Deaver et al., 2018; Hynonen et al., 2016; Png et al., 2010; Wang et al., 2013; Yan et al., 2021). Previous research associated Mediterranean diet adherence in overweight and obese individuals with decreased faecal *R. torques* abundance (Meslier et al., 2020), supporting our associations with Mediterranean dietary pattern and *R. torques* abundance in NHP. However, the function of breast-specific *R. torques* is unknown.

Mucins are large glycoproteins comprising the main structural components of mucus and facilitate interactions between microbes and epithelial surfaces. Mucins display high turnover in the gut, with continuous biosynthesis and degradation to maintain healthy gut homeostasis (Paone and Cani, 2020). Breast tumours also display elevated and aberrant mucin-1 (muc-1) on the cell surface and are associated with poor prognosis (Jing et al., 2019). Several gut bacterial species express the enzymes capable of digesting mucins to free monosaccharides and amino acid residues. These mucin-degrading bacteria, such as *R. torques*, may increase mucin breakdown byproducts, such as free glycan oligosaccharides, fucose, and sialic acid. These metabolites could be detected systemically or may serve as an energy source for other bacterial species, promoting a community microbial shift (Engevik et al., 2021). *N*-acetylneuraminic acid (NANA; Neu5Ac) is the major form of sialic acid in humans. Elevated plasma sialic acid was observed in breast cancer patients (Zhang et al., 2018). Therefore, the elevated *R. torques* in breast of NHP with gut barrier dysfunction or Western diet consumption may promote breast cancer risk. Further studies on the causality between breast and gut-specific *R. torques* abundance and breast tumorigenesis are needed to explore this potential link.

Breast *Ruminococcus torques* and *Ruminococcus flavefaciens* were elevated in NHP subjects with elevated plasma LPS. This may be due, in part, to environmental selection pressures on present breast microbes by elevated LPS presence as lipid A of LPS stimulated growth of lactate-producing bacteria (Dai et al., 2020). However, previous studies demonstrate that a fibre-utilising specific strain, *R. flavefaciens* FD-1 did not significantly respond to LPS in regards to logarithmic growth or short-chain fatty acid production (Dai et al., 2020), potentially refuting this aspect as a contributor to the shift observed in breast *Ruminococcus* abundance in subjects with elevated plasma LPS.

In conclusion, our report highlights the overall independence of the breast microbiome from the gut populations as shown by the minimal overlap in species present in both compartments potentially due to differences in environmental factors. Gut barrier dysfunction, characterised by metabolic endotoxemia, was associated with differences in the breast microbiome regardless of dietary pattern suggesting gut health may influence the breast microbiome. However, the exact mechanism is unknown. Moreover, we show dietary pattern modifies both gut and breast compartments and therefore represents a novel mechanism to target for potential health outcomes.

## Supplementary material

For supplementary material accompanying this paper visit https://doi.org/10.1017/gmb.2022.9. click here to view supplementary material
