# Supplementary material for: Impact of gut permeability on the breast microbiome using a non-human primate model
Source: Gut Microbiome (Camb). 2022 Nov 9;3:e10. doi: 10.1017/gmb.2022.9 (PMC9990890; doi:10.1017/gmb.2022.9)
Supplement: Supplementary file 1 [file S2632289722000093sup001.docx]

**Supplemental Figures and Legends**


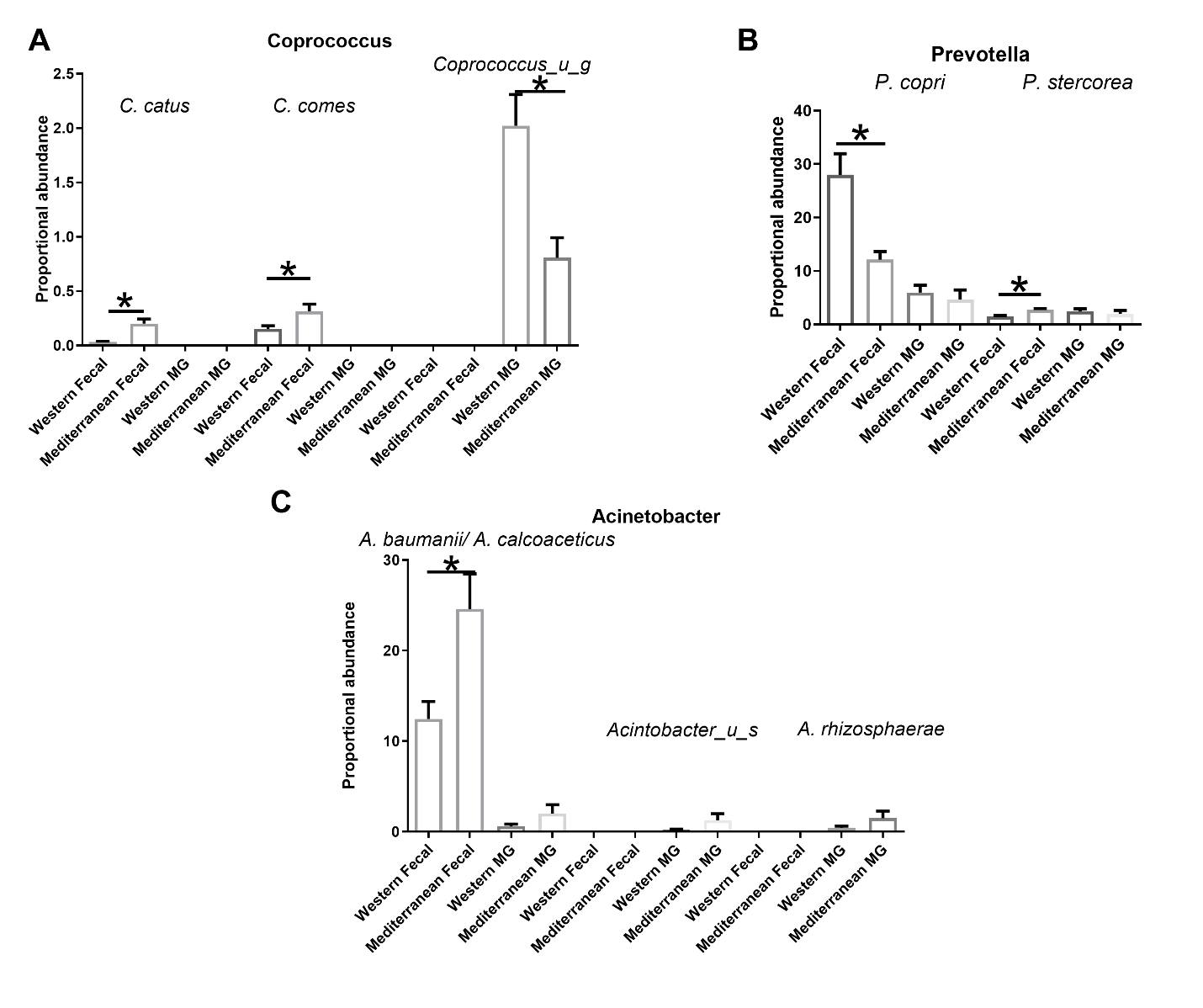


Supplemental Figure S1. A. Species specific localization of Coprococcus in the gut and breast regulated by diet. B. Prevotella copri and Prevotella stercorea are present in both tissue compartments but is only regulated by diet in the gut. C. Species specific localization of Acinetobacter in the gut and breast regulated by diet.
